# Characterizing the (Perceived) Newsworthiness of Health Science Articles: A Data-Driven Approach

**DOI:** 10.2196/medinform.5353

**Published:** 2016-09-22

**Authors:** Ye Zhang, Erin Willis, Michael J Paul, Noémie Elhadad, Byron C Wallace

**Affiliations:** ^1^ Department of Computer Science University of Texas at Austin Austin, TX United States; ^2^ College of Media, Communication and Information University of Colorado Boulder Boulder, CO United States; ^3^ Department of Information Science University of Colorado Boulder Boulder, CO United States; ^4^ Biomedical Informatics Columbia University New York, NY United States; ^5^ College of Computer and Information Science Northeastern University Boston, MA United States

**Keywords:** natural language processing, text classification, press release, media coverage

## Abstract

**Background:**

Health science findings are primarily disseminated through manuscript publications. Information subsidies are used to communicate newsworthy findings to journalists in an effort to earn mass media coverage and further disseminate health science research to mass audiences. Journal editors and news journalists then select which news stories receive coverage and thus public attention.

**Objective:**

This study aims to identify attributes of published health science articles that correlate with (1) journal editor issuance of press releases and (2) mainstream media coverage.

**Methods:**

We constructed four novel datasets to identify factors that correlate with press release issuance and media coverage. These corpora include thousands of published articles, subsets of which received press release or mainstream media coverage. We used statistical machine learning methods to identify correlations between words in the science abstracts and press release issuance and media coverage. Further, we used a topic modeling-based machine learning approach to uncover latent topics predictive of the perceived newsworthiness of science articles.

**Results:**

Both press release issuance for, and media coverage of, health science articles are predictable from corresponding journal article content. For the former task, we achieved average areas under the curve (AUCs) of 0.666 (SD 0.019) and 0.882 (SD 0.018) on two separate datasets, comprising 3024 and 10,760 articles, respectively. For the latter task, models realized mean AUCs of 0.591 (SD 0.044) and 0.783 (SD 0.022) on two datasets—in this case containing 422 and 28,910 pairs, respectively. We reported most-predictive words and topics for press release or news coverage.

**Conclusions:**

We have presented a novel data-driven characterization of content that renders health science “newsworthy.” The analysis provides new insights into the news coverage selection process. For example, it appears epidemiological papers concerning common behaviors (eg, alcohol consumption) tend to receive media attention.

## Introduction

### Background

Health news is an increasingly popular topic in news media [1] and has been shown to improve health outcomes [2,3]. Communicating health science in layman’s terms can often be difficult. Information subsidies, such as press releases, are resources for journalists that mitigate this difficulty by facilitating information transfer. The role of information subsidies and their importance to the development of health news and agenda building is related to the demands of the journalism industry [4].

Gandy [5] first defined *information subsidy* as source information provided to a newsroom, and Berkowitz and Adams [6] further defined subsidy as anything provided to the media in order to gain time or space. Press releases, which are often written by journal staff members in the form of news stories, are one type of information subsidy. To increase the rate of publication, public relations practitioners write press releases with journalistic news values, defined as the elements of a story that make it likely to be published [7]. News values, such as proximity, significance, and novelty, act as criteria for deciding what is newsworthy and most likely to increase audience attention.

In this study, we aim to use data-driven, quantitative approaches to address the following questions: What topical content in health science articles correlates with receiving, or not receiving, a press release? Relatedly, what topical content correlates with receiving, or not receiving, news media coverage? What are the differences in the content of articles covered by the news media versus those that receive a press release?

### Motivation and Related Work

The news media are powerful conduits by which to disseminate important information to the public [8]. There is a chasm between the constant demand for up-to-date information and shrinking budgets and staff at newspapers around the globe. Information subsidies such as press releases are often looked to as a way to fill this widening gap. As a standard of industry practice, public relations professionals generate packaged information to promote their organization and to communicate aspects of interest to target the public [9].

Agenda setting has been used to explain the impact of the news media in the formation of public opinion [10]. The theory posits that the decisions made by news gatekeepers (eg, editors and journalists) in choosing and reporting news plays an important part in shaping the public’s reality. Information subsidies are tools for public relations practitioners to use to participate in the building process of the news media agenda [11,12].

In the area of health, journalists rely more heavily on sources and experts because of the technical nature of the information [12,13]. Tanner [14] found that television health-news journalists reported relying most heavily on public relations practitioners for story ideas. Another study of science journalists at large newspapers revealed that they work through public relations practitioners and also rely on scientific journals for news of medical discoveries [15]. Viswanath and colleagues [4] found that health and medical reporters and editors from small media organizations were less likely to use government websites or scientific journals as resources, but were more likely to use press releases. In other studies, factors such as newspaper circulation, publication frequency, and community size were shown to influence publication of health information subsidies [16-18].

This study focuses on media coverage of developments in health science and scientific findings. Previous research has highlighted factors that might promote press release generation for, and news coverage of, health science articles. This work has relied predominantly on qualitative approaches. For instance, Woloshin and Schwartz [19] studied the press release process by interviewing journal editors about the process of selecting articles for which to generate press releases. They also analyzed the fraction of press releases that reported study limitations and related characteristics. Tsfati et al [20] argued through content analysis that scholars’ beliefs in the influence of media increases their motivation and efforts to obtain media coverage, in turn influencing the actual amount of media coverage of their research.

In this study, we present a complementary approach using data-driven, quantitative methods to uncover the topical content that correlates with both news release generation and mainstream media coverage. Our hypothesis is that there exist specific topics—for which words and phrases are proxies—that are more likely to be considered “newsworthy.” Identifying such topics will illuminate latent biases in the journalistic process of selecting scientific articles for media coverage.

### Contributions

In this work, we apply natural language processing and statistical machine learning techniques to characterize features of scientific articles that receive media coverage. Specifically, we aim to build interpretable statistical models that can reliably predict whether a published health science article will (1) receive a press release from the publishing journal and (2) garner media coverage in mainstream outlets.

To explore these processes empirically we have constructed novel datasets. Our preliminary work [21] showed that one can induce models to reliably discriminate between articles that receive press coverage and those that do not using ”bag-of-words” representations of articles with count variables for unigrams and bigrams extracted from article titles and abstracts—unigrams are single words and bigrams are sequences of two adjacent words. Here we substantially extend this preliminary work as follows:

1. We use supervised latent Dirichlet allocation (sLDA) [22] to uncover discriminative topics that correlate with media attention, in addition to simple n-gram correlations.

2. We analyze a new corpus [23] that contains information concerning both press release issuance and media coverage for all articles it contains. Press releases were issued for all articles in this set, but only a subset garnered media attention, thus providing opportunity to disentangle factors that correlate with each type of press.

Our models are able to reliably discriminate between articles that will and will not (1) motivate a press release and (2) receive media coverage. We report robust predictors for these two tasks, both in terms of words and bigrams in a discriminative bag-of-words framework and with respect to higher-level topics uncovered via sLDA.

## Methods

### Datasets

We now describe the datasets that we have constructed to empirically investigate patterns in press release generation for, media coverage of, and social media attention to, health science articles. We made all of these datasets publicly available, along with our code, to facilitate future research [24].

First, we augmented the dataset recently introduced by Sumner and colleagues [23] in their work addressing the association between exaggeration in health-related science news articles and academic press releases. We will refer to this dataset as Sumner. It contains 462 press releases written for articles published in biomedical and health-related journals by 20 leading UK universities in 2011. For each press release, the authors sourced the corresponding journal article and print or online news stories from national press outlets using the Nexis database, the BBC, Reuters, and Google; the number of news stories per press release ranged from 0 to 10.

Sumner and colleagues coded each journal article, press release, and news piece using a detailed protocol that is available online [25]. We derived two corpora from the Sumner dataset: one was used to investigate press release (PR) issuance, which we call Sumner PR, and the other was used to model news coverage (NC), which we call Sumner NC.

Additionally, we constructed two datasets, Journal of the American Medical Association (JAMA) and Reuters, which we have described in our earlier work [21]. For both of these datasets, we had to generate *negative* instances: health science articles that did not receive media coverage, or for which no press releases were written. To this end, we relied on a novel matched sampling approach [26] aimed at identifying articles that did not garner attention but that had similar characteristics (ie, were published in the same year and in the same journal) to those that did. We describe this process in greater detail below.

We decomposed our aims into distinct modeling tasks to be undertaken using the associated datasets. We treated these as predictive tasks for validation purposes, but our interest is primarily in the predictive features, rather than classifier performance, as such. Table 1 summarizes the four tasks and their corresponding corpora.

**Table 1:** Summary of the four tasks and their associated datasets.

Task	Source	Positive instances in dataset, n (%)	Negative instances in dataset, n (%)	Title length (words), mean (SD)	Abstract length (words), mean (SD)
PR^a^	Sumner PR (N=3024)	422 (13.96)	2602 (86.04)	13 (5)	214 (67)
PR	JAMA^b^ (N=10,760)	846 (7.86)	9914 (92.14)	13 (5)	335 (82)
NC^c^	Sumner NC (N=422)	214 (50.7)	208 (49.3)	14 (5)	226 (79)
NC	Reuters (N=28,910)	1343 (4.65)	27,567 (95.35)	14 (6)	267 (86)

^a^PR: press release.

^b^JAMA: Journal of the American Medical Association.

^c^NC: news coverage.

### Press Release Datasets

#### Sumner Press Release

Our first use of the Sumner corpus [23] involved constructing a dataset to use to induce a discriminative model to predict which scientific articles will receive press releases. To achieve this, we needed to link press releases to the corresponding scientific publications that they cover. For this, we relied on the search functionality in PubMed [27], which provides an interface for searching the over 24 million publications indexed by MEDLINE. We used this to identify the journal articles corresponding to each entry in the Sumner corpus. Specifically, we searched PubMed for the original journal article using the title entered in the coding sheet. In this way, we identified citation information—title, abstract, and Medical Subject Headings (MeSH) keywords—for 422 out of the 460 articles covered by press releases in the Sumner corpus. We were unable to find the remaining 38 articles on PubMed.

**Figure 1:**
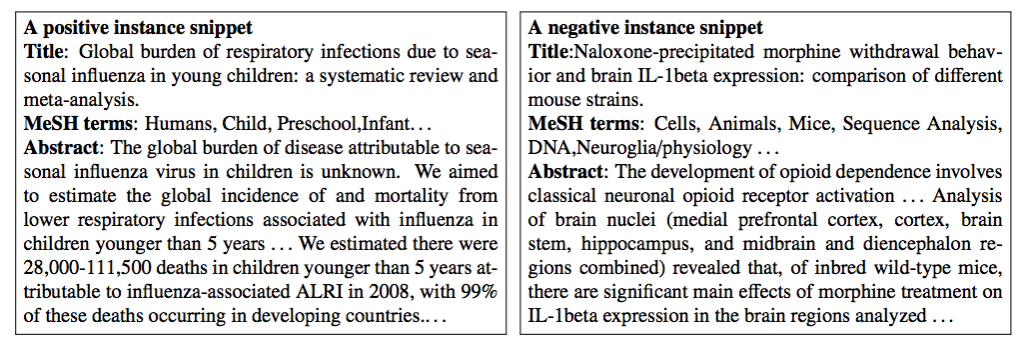
A pair of positive and negative instance snippets from the Sumner press release (PR) dataset.

All 422 of these articles constitute *positive* examples, because all received press releases. We therefore collected *negative* instances via the matched sampling approach, which proceeded as follows. For each citation, we sampled up to 10 articles from the same journal and the same issue for which no press releases were issued. Our aim in so doing was to isolate content predictors that correlate with garnering media attention, independent of publication venue and temporal factors. In total, we retrieved 2602 citations using this approach. Figure 1 depicts a pair of positive and negative snippets.

#### Journal of the American Medical Association

The JAMA corpus comprised 846 positive instances, defined as articles for which journal editors created a press release—all journals in this corpus belong to the JAMA network [21]. Negative instances were again selected via matched sampling, focusing on articles from the same journal and year, but for which no press release was issued. After removing duplicates, this corpus comprised 9914 *negative* articles. This collection was exhaustive, containing all press releases available on the JAMA Web archive from October 1, 2012, to October 1, 2014.

### News Coverage Datasets

#### Sumner News Coverage

For the first news coverage prediction task, we used the 422 articles contained in the Sumner dataset. In this case, we knew which articles were covered by one or more news outlets, and we could therefore derive positive and negative labels for each article. In all, 214 of these articles received news media coverage. We will refer to this dataset as Sumner NC.

#### Reuters

The Reuters corpus [21] comprised health news stories that reported on particular biomedical and health research studies published by the Reuters news agency. In each story, Reuters journalists cited and linked to the original scientific article on which the story reported. Thus, the Reuters stories and their corresponding scientific articles provided us with *positive* instances for the media coverage prediction task. We again used our matched sampling method to sample up to 20 articles for each positive instance as described in Wallace et al [21]. Briefly, we sampled citations published in the same journal, year, and volume as *positive* instances. This resulted in 1343 positive instances and 27,567 negative instances.

### Machine Learning Algorithms

#### Overview

In this section, we describe the machine learning methods we used to analyze the corpora. Broadly, these can be decomposed into our discriminative learning approach and the generative supervised topic modeling method we used to uncover latent topics that correlate with newsworthiness.

#### Discriminative Learning

**Figure 2:**
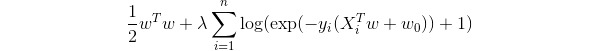
Equation 1. *X*_i_ is the feature vector representing the *i* th article—comprising counts of uni- and bigrams— *y*_i_ is the label for this article, *w* is the weight vector to be estimated from the data, and *w*is an intercept term. λ is a scalar hyper-parameter that controls the trade-off between regularization strength and empirical predictive performance on the training set.

For discriminative learning, we used standard logistic regression with a squared ℓ2 norm penalty placed on the weights for regularization. Specifically, given a labeled corpus, we optimize the objective in Equation 1 in Figure 2. In Figure 2, *X*_i_ is the feature vector representing the *i* th article—comprising counts of uni- and bigrams— *y*_i_ is the label for this article, *w* is the weight vector to be estimated from the data, and *w*_0_ is an intercept term. We fit this model using LIBLINEAR (Machine Learning Group at National Taiwan University) [28]. λ is a scalar hyper-parameter that controls the trade-off between regularization strength and empirical predictive performance on the training set. We performed five-fold cross-validation and reported average area under the curve (AUC) scores. Cross-validation is a standard means of assessing model performance in which one splits the data into *k* disjoint “folds” (here *k*=5) and holds one out at a time. The model is then trained using *k*-1 folds, and performance metrics are calculated on the held-out fold. This process is repeated *k* times, resulting in *k* estimates of performance. Here we used the AUC metric, which is a widely used measure of classifier discriminative performance that captures the probability that a given positive instance will be ranked above an arbitrary negative instance by the model. To select the λ hyper-parameter (Equation 1), we performed a logarithmic line search over possible values ranging from 0.00001 to 100—smaller λ values correspond to stronger regularization. We kept the value that maximized average performance, as assessed via nested cross-validation; thus, we performed λ selection independently for each fold, as this was tuned on the available training data.

As features in the logistic regression model, we used uni- and bigrams extracted from titles, abstracts, and MeSH terms. MeSH terms are Medical Subject Headings drawn from a controlled vocabulary maintained by the National Library of Medicine (NLM). These are manually assigned to citations by trained annotators at the NLM.

For text preprocessing, we used a standard English stop word list, and only kept features that appeared in at least two instances in a given dataset. We kept, at most, the 50,000 most frequently occurring features in the datasets, in cases where there were more than 50,000 unique features. The numbers of features for each task are summarized together with the sLDA model in the next section.

To identify robustly predictive features, we used bootstrap sampling to construct confidence intervals around coefficient point estimates. Specifically, we fit a regularized logistic regression model to each bootstrap training sample and recorded estimated coefficient values for each feature. We repeated this process 1000 times, deriving a variance from the observed estimates. We then constructed an approximate 95% confidence interval around coefficients using the normal approximation method [29].

#### Supervised Topic Modeling

Statistical topic models have emerged as an important tool for discovering topics from large collections of text documents. Topic models postulate a *generative story,* in which each document comprises a mixture of topics and each topic corresponds to a probability distribution over words. This is the model specified by latent Dirichlet allocation (LDA) [30].

Supervised topic modeling is a variant of this, in which auxiliary meta-data about documents (ie, supervision) is assumed to be available [31]. Typically, this supervision is expressed as labels or tags on documents. In sLDA, one then assumes a model similar to that of standard LDA: a document is again associated with a distribution over topics that are in turn modeled as distributions over words. However, sLDA extends this to additionally model the document attributes (ie, labels), conditioned on estimated topic frequencies. In our case, the label for a given document was whether or not it received a press release or media coverage—we model these separately. Thus, we aimed to uncover topics that explicitly correlated with press release issuance and media coverage.

**Figure 3:**
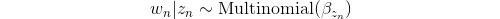
Word distribution. w_n_ is the word at position n, z_n_ is the topic at position n, and β_K_ is a vector of term probabilities.

**Figure 4:**
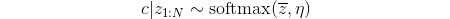
Class label. c is class label, η is a K-dimensional vector of real values.

**Figure 5:**
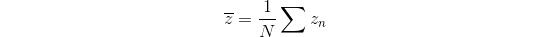
Empirical topic frequencies.

**Figure 6:**
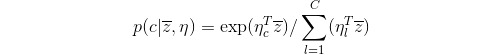
Equation 2: softmax function.

More specifically, we assumed that there are *K* topics in the corpus, and the number of class labels is *C*. The model parameters are as follows: the *K* topics β_1:K_ (each β_K_ is a vector of term probabilities), the Dirichlet hyper-parameter α, and a set of prediction coefficients for each class *c*. Each coefficient *η*_c_ is a *K*-dimensional vector of real values. The process for generating an article and its label is then modeled as follows:

1. Draw topic proportions θ~Dirichlet(α).

2. For each word in position *n* in the article,

(a) Draw topic assignment z_n_|θ ~ Multinomial (θ)

(b) Draw word as in Figure 3.

3. Draw class label as in Figure 4, where *N* is the total number of words in the article, and the empirical topic frequencies of the article is as shown in Figure 5. The softmax function is as shown in Equation 2 in Figure 6.

Here, the labels *c* for each article are binary: they either received a press release or media coverage, or did not. For parameter estimation, we used the approximate inference algorithm presented in Wang et al [31]. We set the number of topics *K* to 20, which we viewed as an intuitively reasonable number of topics to assume. We set the symmetric Dirichlet prior α to 1.

The words comprising our vocabulary were unique unigrams extracted from citation titles, abstracts, and MeSH terms. We again kept up to 50,000 of the most frequently occurring words in the dataset as features. Ultimately, for the discriminative task—for which we used logistic regression—we used the following: 50,000 features for Sumner PR; 50,000 for JAMA; 10,004 for Sumner NC, which is much smaller; and 50,000 features for the Reuters corpus. For generative modeling (ie, using sLDA), we are left with the following: 23,561 features for the Sumner PR dataset; 23,539 for the JAMA dataset; 5796 for Sumner NC; and 50,000 for the Reuters corpus.

## Results

### Press Release Issuance

#### Sumner Press Release

With respect to discriminating between articles that did and did not receive a press release in the Sumner PR dataset, we achieved a mean AUC of 0.666 (SD 0.019; range 0.636-0.720 across five folds of cross-validation), indicating relatively strong predictive performance. We report the top 25 most robustly predictive n-gram features— negative and positive—in Textboxes 1 and Textboxes 2. To extract the features that are consistently correlated with positive instances, we ranked the predictors in descending order according to the lower bound of their corresponding confidence intervals, which were derived via bootstrap estimation as discussed above. Similarly, for negative features, we sorted predictors in ascending order of estimated confidence interval upper bounds.

**Figure 7:**
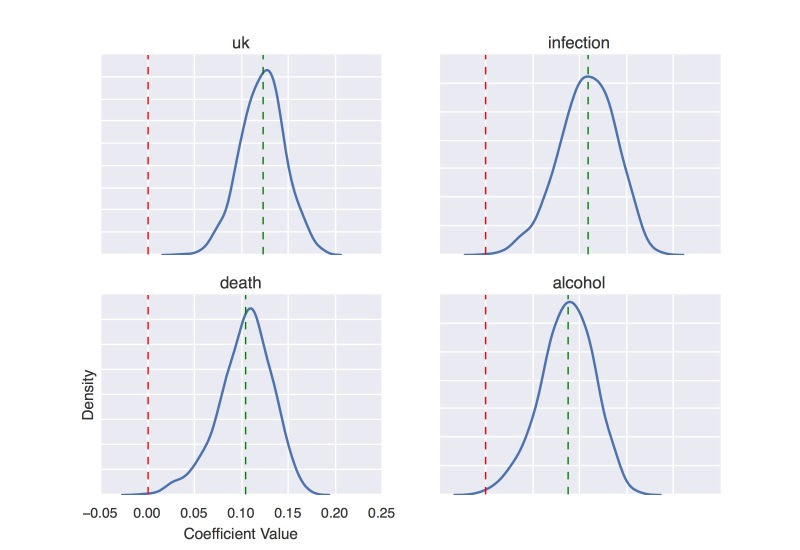
Density curve of coefficient values of four positively predictive words on Sumner press release datasets.

**Figure 8:**
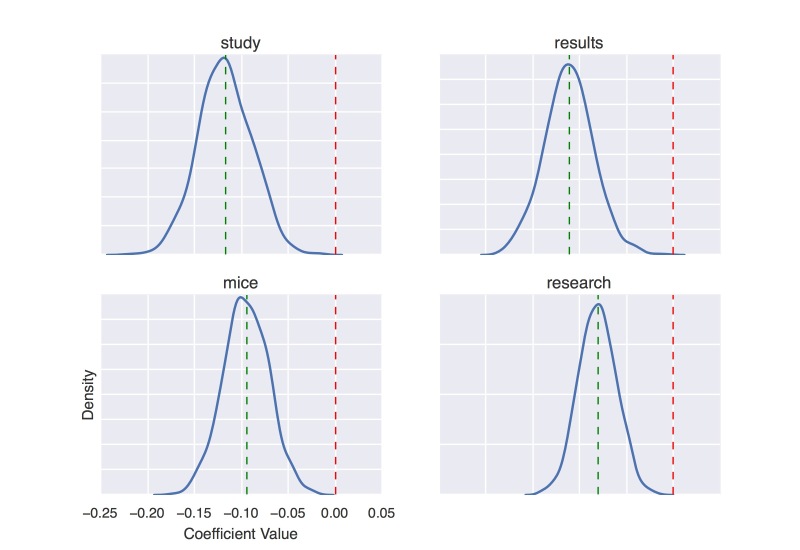
Density curve of coefficient values of four negatively predictive words on Sumner press release datasets.

Figures 7 and 8 show coefficient value distributions, as constructed via the bootstrap, for selected features that positively and negatively correlate with press release issuance for articles in Sumner PR dataset, respectively.

**Figure 9:**
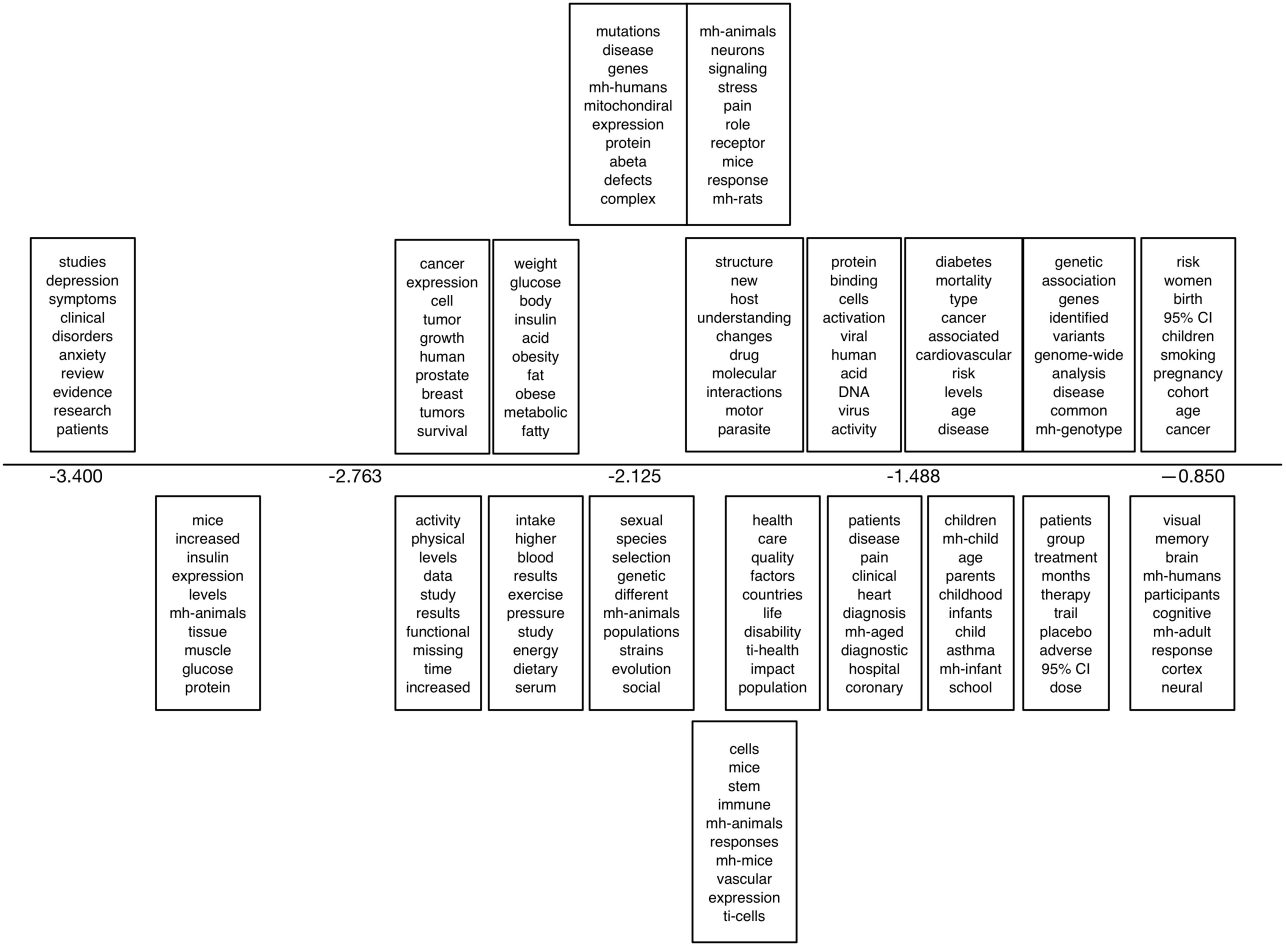
The top 10 most probable words under the topics uncovered by a 20-topic supervised latent Dirichlet allocation model fit to the Sumner press release dataset. The horizontal axis corresponds to the coefficient of the topic. mh: this prefix indicates a Medical Subject Headings (MeSH) term; ti: this prefix indicates a title term.

We also present output from a 20-topic sLDA model fit to the Sumner PR dataset in Figure 9. The horizontal axis corresponds to the coefficient of the topic, capturing correlation with press release issuance: topics with larger values, toward the right end of the plot, are therefore correlated with press releases being issued (ie, these topics are more likely to appear in articles that receive press releases). We report the top 10 most probable words estimated for each topic. Here we used whether or not an article received a press release as a label.

**Top 25 negative features of press release prediction on Sumner press release dataset, ranked by upper bound of confidence interval.**

Top 25 negative features:

- decreased
- study
- results
- sexual
- mice
- based
- research
- signaling
- evaluated
- regarding
- proposed
- protein
- states
- discuss
- program
- various
- lesions
- review
- thyroid
- analyzed
- performed
- overexpression
- medical
- asd

**Top 25 positive features of press release prediction on Sumner press release dataset, ranked by lower bound of confidence interval.**

Top 25 positive features:

- uk
- england
- infection
- death
- MH-great-britain (MH prefix indicates a Medical Subject Headings [MeSH] term)
- magnetic
- alcohol
- life
- weeks
- MH-england/epidemiology
- magnetic resonance
- setting
- main outcome
- pregnancy
- british
- functional magnetic
- increase
- resonance imaging
- MH-great-britain MH-humans
- resonance
- TI-study (TI prefix indicates a title term)
- adjusted
- brain
- outcomes

**Figure 10:**
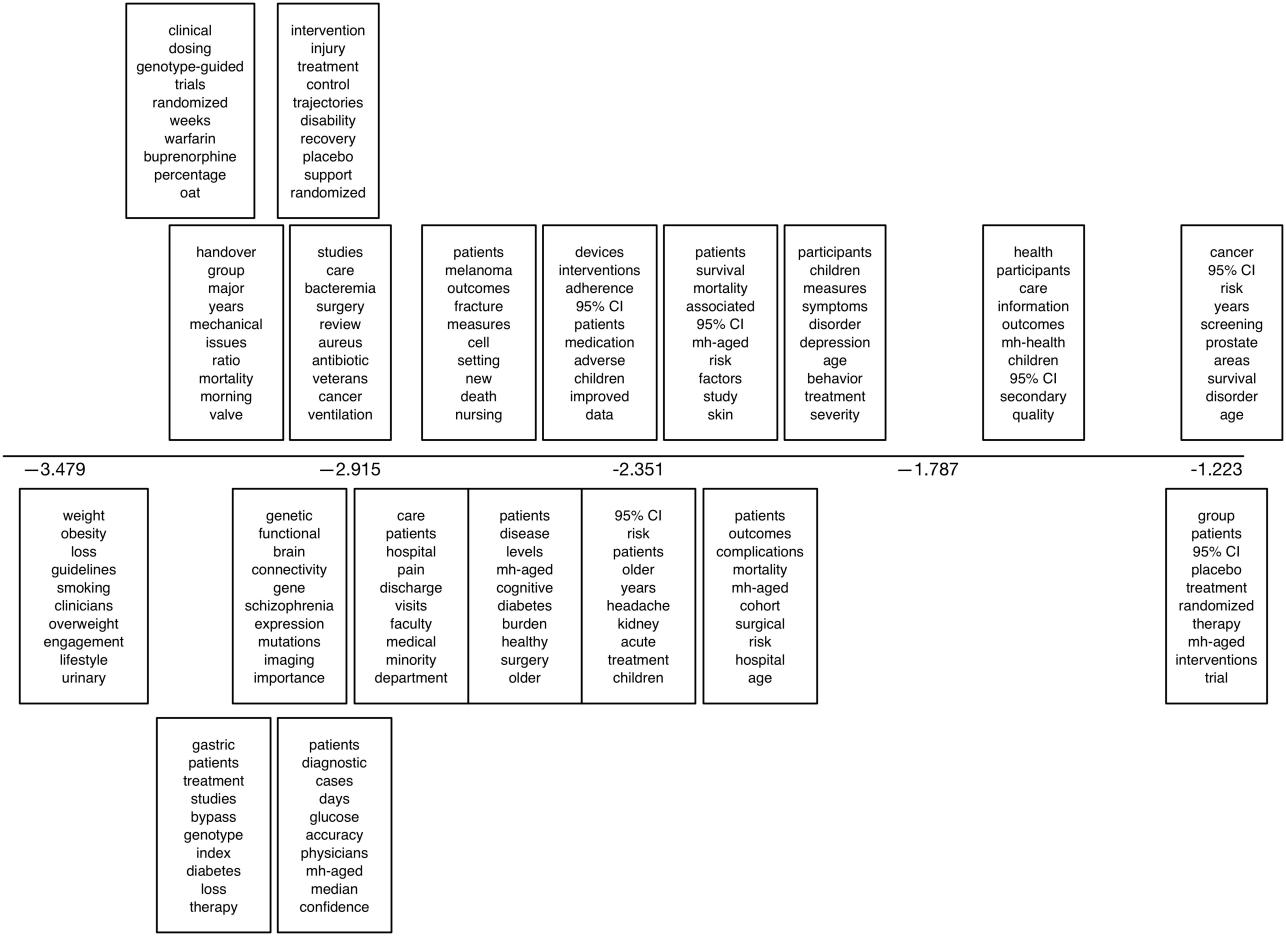
The top 10 most probable words under the topics uncovered by a 20-topic supervised latent Dirichlet allocation model fit to the Journal of the American Medical Association dataset, using press release issuance as the supervision. The horizontal axis corresponds to the coefficient of the topic. mh: this prefix indicates a Medical Subject Headings (MeSH) term.

#### Journal of the American Medical Association

The analysis reporting informative features for logistic regression prediction was presented in our preliminary work [21], so we do not repeat this here. However, we note that the mean AUC score attained on this dataset was 0.882 (SD 0.018; range 0.853-0.918 across five folds). Figure 10 shows the 20-topic sLDA model fit to this dataset, again using press release issuance as the supervision.

### News Coverage Results

#### Sumner News Coverage

**Figure 11:**
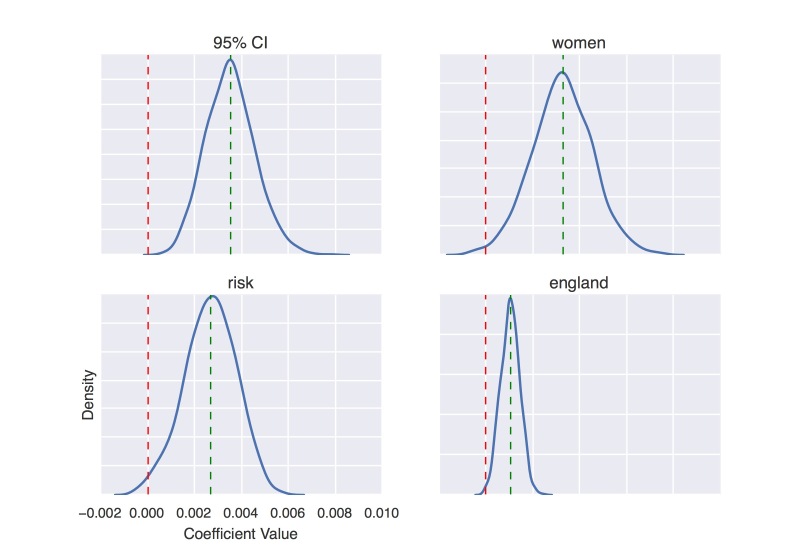
Density curve of coefficient values of four positively predictive words on Sumner news coverage (NC) dataset.

**Figure 12:**
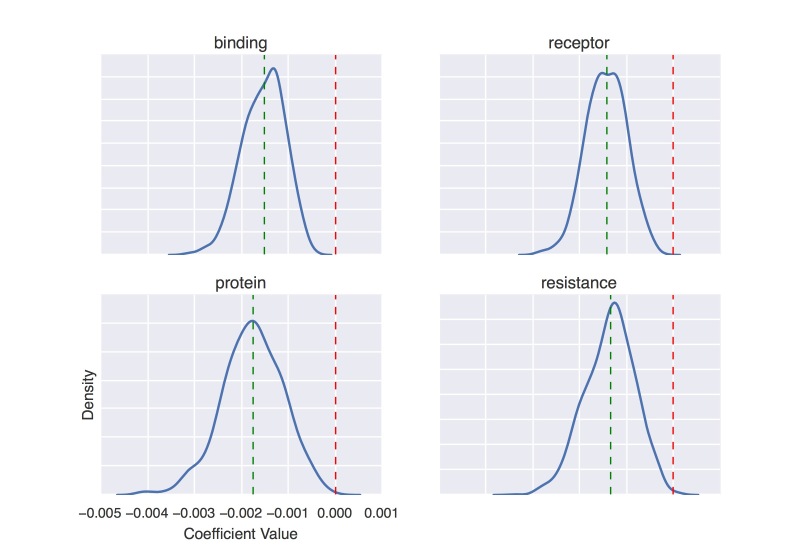
Density curve of coefficient values of four negatively predictive words on Sumner news coverage (NC) dataset.

On the Sumner NC dataset, we experimented with two different feature sets: (1) features extracted from the journal articles and (2) features extracted from the corresponding press release text. Our model using journal features achieved a mean AUC of 0.591 (SD 0.044) and ranged from 0.502 to 0.701 across five folds; our model using press release features achieved a mean AUC of 0.575 (SD 0.023) and ranged from 0.497 to 0.622. We note that this exhibits weaker correlation than press release prediction, although it is still better than chance (ie, 0.5). We report the top 25 most predictive features (ie, terms) of news coverage for each feature set in Textboxes 3-6. We rank the features using the same method as in Textboxes 1 and Textboxes 2. In Figures 11 and 12, we show the density curves of coefficient values of four positively predictive words and four negatively predictive words, respectively.

**Top 25 negative features for news coverage prediction on the Sumner news coverage (NC) corpus using the original article abstracts/titles as features.**

Top 25 negative article features:

- binding
- receptor
- development
- protein
- resistance
- identify
- surface
- MH-molecular-sequence-data (MH prefix indicates a Medical Subject Headings [MeSH] term)
- direct
- responses
- disruption
- rapidly
- domain
- regions
- structure
- synaptic
- TI-early (TI prefix indicates a title term)
- MH-models-biological
- specific
- using
- culture
- MH-amino-acid-sequence
- TI-children
- understanding
- complexes

**Top 25 negative features for news coverage prediction on the Sumner news coverage (NC) corpus using the original press releases as features.**

Top 25 negative press release features:

- resistance
- physical
- proteins
- childhood
- liverpool
- understanding
- born
- impact
- design
- opportunities
- university
- attention
- american
- bristol published
- sentences
- changes
- university birmingham
- discovered
- options
- published
- cardiac
- revealed
- date
- leave
- led professor

**Top 25 positive features for news coverage prediction on the Sumner news coverage (NC) corpus using the original article abstracts/titles as features.**

Top 25 positive article features:

- 95% ci
- women
- use
- risk
- countries
- variation
- hazard
- body
- england
- participants
- TI-cohort (TI prefix indicates a title term)
- council
- TI-cohort TI-study
- research council
- medical research
- individual
- individual
- sex
- main outcome
- cohort study
- systolic blood
- relevant
- cancers
- research
- TI-gene

**Top 25 positive features for news coverage prediction on the Sumner news coverage (NC) corpus using the original press releases as features.**

Top 25 positive press release features:

- face
- research funded
- shown
- better
- motor
- years
- gene
- england
- edinburgh
- trial
- production
- flu
- council
- targeting
- producing
- roslin
- research council
- roslin institute
- widespread
- royal society
- faces
- providing
- website
- affecting
- exciting

**Figure 13:**
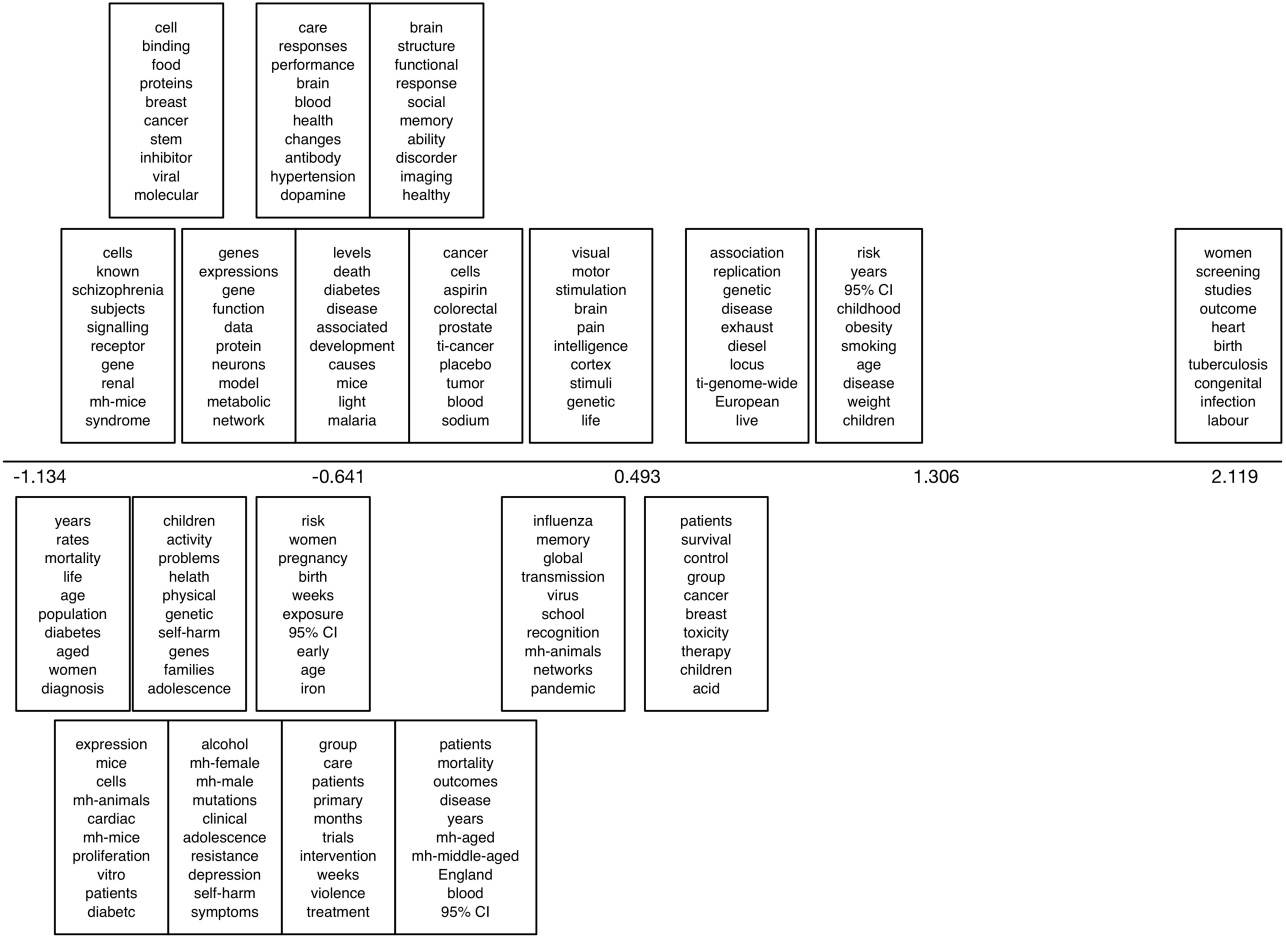
Top 10 most probable words in the topics uncovered by the supervised latent Dirichlet allocation model—again assuming 20 topics—fit to the Sumner news coverage dataset. mh: this prefix indicates a Medical Subject Headings (MeSH) term; ti: this prefix indicates a title term.

In Figure 13, we show results from the sLDA model fit to the Sumner NC corpus using journal features. The plot is as described above, only the horizontal axis now captures the relative correlation with news coverage estimated for each topic. Here, the supervision captured whether articles received news coverage or not and, hence, we can see which topics (anti)correlate with this.

#### Reuters

**Figure 14:**
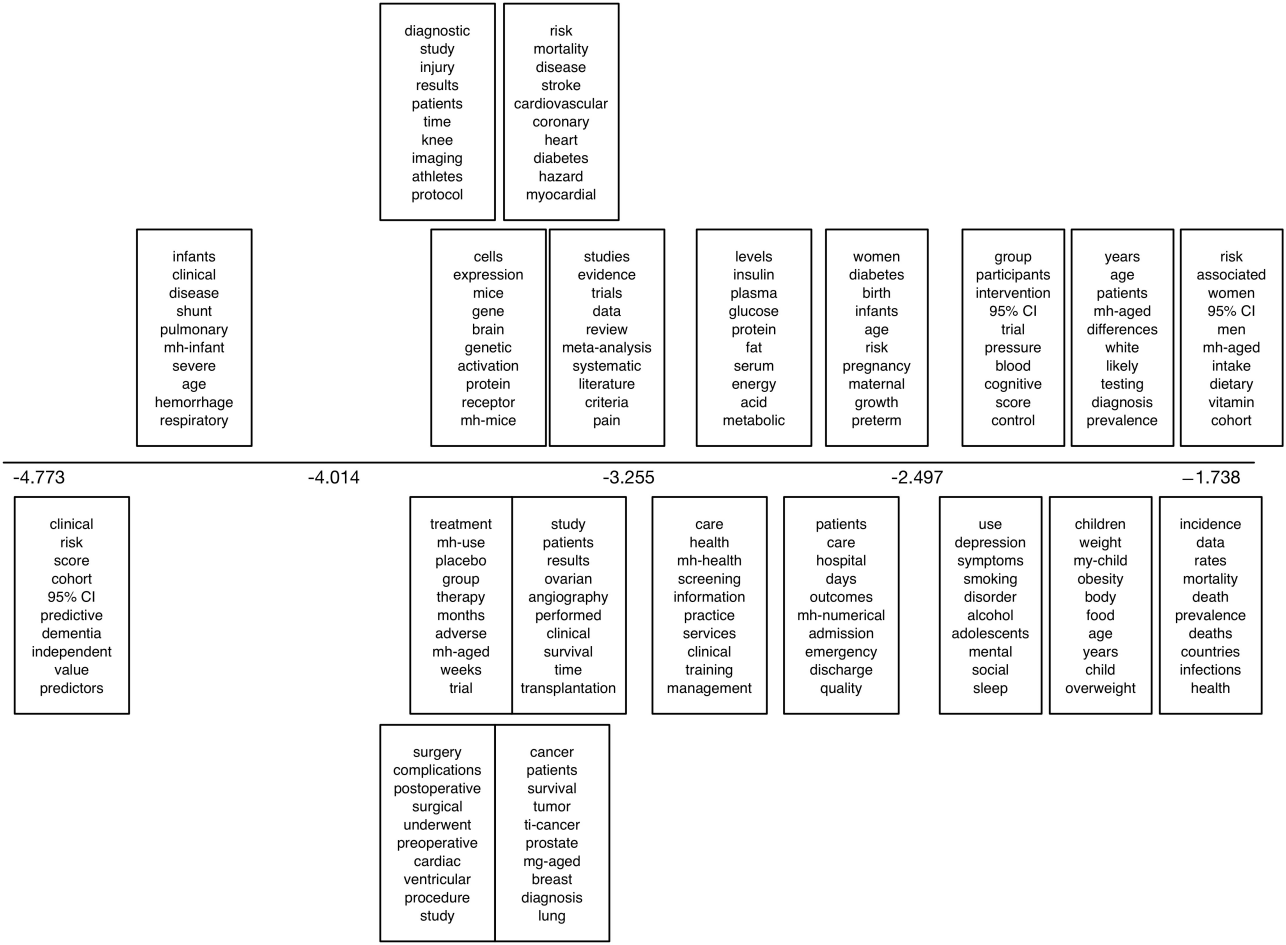
Top 10 words from the 20 topics uncovered by the supervised latent Dirichlet allocation on the Reuters corpus, again using news coverage as the supervision. mh: this prefix indicates a Medical Subject Headings (MeSH) term; ti: this prefix indicates a title term.

For the discriminative learning task for this dataset, we have reported results previously [21] and do not repeat them here. Briefly, the mean AUC achieved for this task was 0.783 (SD 0.022; range 0.746-0.811). In Figure 14, we report output from the sLDA model: uncovered topics and their degree of correlation with news media coverage. Inspecting the topics suggests a fair amount of overlap with the discriminative topics in Figure 7.

## Discussion

As news organizations weather the fast-changing information landscape, press releases are now assisting journalists to fill news holes, more than ever before [32]. News organizations are increasingly eschewing the use of specialist reporters such as health science journalists, opting instead to rely on sources and experts [8]. This shift has enabled companies and organizations to play a role in setting the news agenda.

In our prior preliminary work [21], we reported that words such as *women*, *95% CI*, and *drinking* were predictive of both press release issuance and media coverage. Presumably, this reflects interest in population-level results that relate to issues of common concern. Meanwhile, features anticorrelated with press release generation and media coverage seem to be indicative of basic sciences work (eg, binding, receptor, and mice).

This study examined the topics covered by press releases generated by scientific journals. Specifically, we have presented new corpora, methods, and results that aim to illuminate factors that correlate with press release generation for, and news media coverage of, health science articles. Our analysis indicates that scientific journals intentionally disseminate press releases that cover topics likely to be found “newsworthy” by lay audiences. For example, the flu was a topic frequently found in articles deemed newsworthy and in those for which journal editors wrote press releases.

Some of the press release topics were very general and applicable to broad audiences. For example, *women* was a word found frequently in articles that received press releases; indeed, *pregnancy* and *women* were among the most probable words under the topic most strongly correlated with press release issuance (see Figure 4). It is intuitive that most audiences would be interested in research related to women, and pregnancy specifically. By selecting topics that are relevant and applicable to general audiences, scientific journals are helping journalists build the news agenda and educate audiences on (sometimes) difficult and complex topics. Scientific journals are selecting specific studies assumed to be newsworthy by the gatekeepers of the news media, working to form public opinion about a topic. Furthermore, because scientific research is often quite complex, scientific journals may be selecting research studies that are both relatable and easier to translate to a lay audience.

There are several practical implications regarding the results of this study. For instance, press releases from scientific journals might be considered a trustworthy source for journalists working in health news. However, journalists should be aware of the limited scope of the breadth of topics covered in press releases and that other research findings should be explored for news coverage.

This research is not without limitations, however. We can only surmise as to why press releases were written on certain health science research findings or why a press release garnered news coverage. More research needs to be conducted on why certain health science articles are chosen as newsworthy and why journalists reported on the research findings they did. Although news values are meant to guide journalists’ selection of news, there are some who argue that news values are broad and vary greatly among news organizations [33]. Based on the methodology used in this paper, it is also a limitation that no further insight was gleaned from the specific press releases or that the media coverage was not examined. More research must be conducted on how health science findings are being explained in press releases, and how media are translating the press releases into news stories.

Moving forward, we are encouraged by our positive results, and believe our models could be improved further in future work. For example, we could move beyond simple lexical features like n-grams and MeSH terms, including high-level concepts as features, such as the size and composition of the study cohort or the affected population, the type of study (eg, observational or controlled), and whether the research is basic or more applied. Richer linguistic features would also be interesting to incorporate, to help understand if certain writing styles are associated with more or less press coverage. When predicting media coverage, it would also be interesting to use features extracted from the press releases in addition to, or instead of, the features from the original journal articles, to understand how press releases influence the news media.

## Abbreviations

AUC: area under the curve
JAMA: Journal of the American Medical Association
LDA: latent Dirichlet allocation
MeSH: Medical Subject Headings
MH/mh: MeSH terms
NC: news coverage prediction task
NLM: National Library of Medicine
PR: press release prediction task
sLDA: supervised latent Dirichlet allocation
Sumner NC: Sumner's dataset for news coverage prediction task
Sumner PR: Sumner's dataset for press release prediction task
TI/ti: title terms
